# Anti-Atherosclerotic Effect of Gossypetin on Abnormal Vascular Smooth Muscle Cell Proliferation and Migration

**DOI:** 10.3390/antiox10091357

**Published:** 2021-08-26

**Authors:** Hui-Hsuan Lin, Ming-Chang Hsieh, Chi-Ping Wang, Pei-Rong Yu, Ming-Shih Lee, Jing-Hsien Chen

**Affiliations:** 1 Department of Medical Laboratory and Biotechnology, Chung Shan Medical University, Taichung City 40201, Taiwan; linhh@csmu.edu.tw (H.-H.L.); cshb183@csh.org.tw (M.-C.H.); cshb015@csh.org.tw (C.-P.W.); 2 Clinical Laboratory, Chung Shan Medical University Hospital, Taichung City 40201, Taiwan; 3 Department of Nutrition, Chung Shan Medical University, Taichung City 40201, Taiwan; smile11619@gmail.com

**Keywords:** gossypetin, atherosclerosis, vascular smooth muscle cells, proliferation, migration, cell-cycle, reactive oxygen species

## Abstract

Gossypetin (GTIN), known as 3,5,7,8,3′,4′-hexahydroxyflavone, has been demonstrated to exert anti-atherosclerotic potential against apoptotic injury in oxidized low-density lipoprotein-incubated endothelial cells, and atherosclerotic lesions of cholesterol-fed rabbits. However, the effect and underlying mechanism of GTIN on abnormal vascular smooth muscle cells (VSMCs) proliferation and migration, a major event in the pathogenesis of atherosclerosis, is still unknown. In this study, non-cytotoxic doses of GTIN abolished the VSMCs A7r5 proliferation and cell-cycle S phase distribution. The GTIN-arrested G0/G1 phase might be performed by increasing the expressions of phosphorylated p53 and its downstream molecules that inhibit the activation of cyclin E/cyclin-dependent kinase (cdk)-2, blocking retinoblastoma protein (Rb) phosphorylation and the subsequent dissociation of Rb/transcription factor E2F1 complex. In addition, the results indicated that GTIN inhibited VSMCs wound-healing and migratory abilities through reducing matrix metalloproteinase (MMP)-9 activity and expression, as well as down-regulating protein kinase B (PKB)/nuclear factor-kappaB (NF-κB) signaling. GTIN also revealed potential in diminishing reactive oxygen species (ROS) generation. These findings suggested the inhibitory effects of GTIN on VSMCs dysfunction could likely lead to the containment of atherosclerosis and other cardiovascular illness.

## 1. Introduction

Gossypetin (GTIN) is a natural hydroxylated flavone with extensive antioxidant, antimicrobial, anti-inflammation, and anti-tumor activities [1]. Previous studies have indicated that GTIN might be a multifunctional agent that performs its activities by diminishing N-methyl-N′-nitro-N′-nitrosoguanidine (MNNG) mutagenicity [2], exerting antioxidant and anti-atherosclerotic effects [3,4], possessing anti-microbial property [5,6], and ameliorating gamma radiation-mediated DNA damage [7], as well as inducing apoptotic and autophagic cell death of cancer cells [8]. As regards the literature of anti-atherosclerosis, GTIN has been shown to be quite effective not only in the inhibition of low-density lipoprotein (LDL) modification, macrophage uptake, and subsequent foam cell formation, but in the promotion of cholesterol efflux [3]. A recent study noted that GTIN effectively suppressed atherosclerotic pathogenesis by exhibiting the protective effects against oxidized LDL-induced oxidative endothelial dysfunction [4]. Regardless of the beneficial effect of GTIN on atherosclerosis, it still exists to be elucidated whether the GTIN’s other action is related to the protective role on vascular smooth muscle cells (VSMCs).

VSMCs dysfunction, including abnormal cell proliferation and migration, is a major event in the atherosclerotic process. In the pathogenesis of atherosclerosis, VSMCs progressively proliferate and migrate from vascular media to neointima with plentiful amounts of extracellular matrix (ECM) proteins, ultimately leading to the plaque formation [9]. In clinics, angioplasty-induced VSMCs dysfunction may be a vital determinant factor in vascular remodeling and restenosis [10]. Previous studies have explored that reactive oxygen species (ROS)-modulated oxidative stress accelerates the development of VSMCs dysfunction, containing ECM remodeling and cell-cycle progression [11,12]. VSMCs proliferation contributes to cell phenotypic remodeling [13]. Upon stimulation of vascular injury, VSMCs dedifferentiate and divide in reactive to mitogens, which are accompanied by progressing the DNA synthesis (S) phase of cell-cycle [14]. The hyperphosphorylation of retinoblastoma (Rb) protein and its release of the key transcription factor E2F1 are hallmarks of the G1 to S transition; otherwise the dephosphorylated Rb protein can bind to the E2F1, blocking the cell to enter S phase [14]. Cyclin D1 or E working in conjunction with its activating subunit cyclin-dependent kinase (cdk)-4 or -2 provides the cell-cycle transitions [15]. The enzyme activities of these cyclin/cdk complexes are downregulated by cdk inhibitors (CDKIs), such as inhibitors of cdk-4 (Ink4) locus, encoding the p15 and p16 proteins and cdk interacting protein/kinase inhibitor protein (Cip/Kip) locus encoding the p21 and p27 proteins, and their upstream factor p53, the cell-cycle gatekeeper [16].

Many studies have provided mechanistic insights such as the findings that an increase in matrix metalloproteinase (MMP) activity results in ECM degradation [17], facilitating VSMCs migration [18]. The MMPs gene expression is vitally modulated at the transcriptional level by nuclear factor-kappaB (NF-κB) or/and activator protein-1 (AP-1) via protein kinase B (PKB, so-called Akt) or extracellular signal-regulated kinase (ERK)-mediated signaling pathways, and at the protein level through their activators or inhibitors [9,19]. Therefore, MMPs and their association with signaling factors are considered as potential therapeutic targets for atherosclerosis [20].

In the previous study, an in vivo model of cholesterol-induced atherosclerotic rabbits and an in vitro oxidized LDL-stimulated oxidative injury in endothelial cells were used to demonstrate that GTIN possesses the anti-atherosclerotic potential [4]. Whether the GTIN could exert the protective effect on VSMCs dysfunction will be further investigated.

## 2. Materials and Methods

### 2.1. Cell Culture and Treatment

Rat aortic VSMCs A7r5 were cultured in Dulbecco’s modified Eagle’s medium (DMEM) supplemented with 10% fetal bovine serum (FBS), 1% L-glutamine, 1.5 g/L sodium bicarbonate (NaHCO_3_), and 1% penicillin-streptomycin at 37 °C in a humidified atmosphere with 5% carbon dioxide (CO_2_). In all experiments, A7r5 cells were pre-cultured in DMEM supplemented with 0.5% FBS for 48 h, as a starvation condition. This condition triggers the cells at resting phase (G0 phase), a period in the cell-cycle in which VSMCs exist in a quiescent contractile phenotype [21], and the starved cells were then treated with 10% FBS in the absence or presence of the indicated doses of GTIN, obtained from ChromaDex Inc. (St. Santa Ana, CA, USA; purity ≥ 99.0%) for additional 48 h.

### 2.2. 3-(4,5-Dimethylthiazol-zyl)-2,5-Diphenyltetrazolium Bromide (MTT) Method

GTIN cytotoxicity toward to A7r5 cells was achieved utilizing MTT method as described in detail elsewhere [22]. In brief, cells were seeded in 24-well plates (10^5^ per well) and treated with 0, 1, 5, 10, 25, 50, 100, and 250 μM GTIN for 48 h and MTT test (metabolic activity) was then analyzed. The concentrations of 1, 5, 10, and 25 μM GTIN in a 48-h treatment was selected for further analysis.

### 2.3. Cell Growth Assay

The growth curve of A7r5 cells (untreated and GTIN-treated cells) were detected using blue exclusion assay, as described by Chou et al. (2019) [23]. The cells were seeded in 10-cm dishes and measured for cell survival each 24 h over a 3-day period. On the day of experiment, the cells were added to 5 mL phosphate buffered saline (PBS) and gently scraped from the dish surface. In order to remove clumps, the PBS volume was thoroughly mixed, and the mixture was centrifuged at 1200 rpm for 5 min to pellet cells. The supernatant was decanted, and then the pellet was re-suspended and mixed thoroughly with trypan blue. After staining, an aliquot was loaded onto a glass counting chamber to count live/dead cells, represented as cell number.

### 2.4. Immunocytochemistry (ICC)

A7r5 cells planted in 6-well plates were fixed with 4% paraformaldehyde for 10 min and permeabilized with 0.1% Triton-X-100 for additional 10 min. For polymerized F-actin staining, the cells were stained with tetramethylrhodamine (TRITC)-phalloidin dye (Sigma-Aldrich, St Louis, MO, USA) for 1 h at room temperature. Cell nuclei were counterstained with 1 mg/mL 4′,6-diamidino-2-phenylindole (DAPI). Images were obtained by fluorescence microscopy.

### 2.5. Cell-Cycle Analysis

After A7r5 cells were treated with or without GTIN, the percentage of cells in the subG1, G0/G1, S, and G2/M phases of cell-cycle was analyzed applying Muse™ Cell Analyzer and Muse™ Cell Cycle Kit (Merck Millipore Ltd., Darmstadt, Germany) depending to the manufacturer’s instructions, as described previously [24].

### 2.6. Western Blotting (WB)

The total protein lysate was extracted by radioimmunoprecipitation assay (RIPA) buffer and protease inhibitors. Bicinchoninicacid (BCA) detection method was utilized to determine the protein concentration. The total amount of protein (50 μg) was denatured and added to the prepared 8–15% gels of sodium dodecyl sulfate-polyacrylamide gel electrophoresis (SDS-PAGE) for electrophoretic separation and transferred to nitrocellulose papers (NC papers). To avoid nonspecific binding, the NC paper was then treated with 5% skim-milk for 1 h. Next, the paper was incubated overnight with diluted p-p53 (sc-135772, Santa Cruz), p53 (sc-6243, Santa Cruz), p27 (sc-1641, Santa Cruz), p21 (sc-6242, Santa Cruz), p16 (sc-28260, Santa Cruz), E2F1 (sc-251, Santa Cruz), p-Rb (#9308s, Cell signaling), Rb (sc-28260, Santa Cruz), MMP-2 (sc-10736, Santa Cruz), MMP-9 (sc-393859, Santa Cruz), p-Akt (#4051s, Cell signaling), Akt (AF6261, Affinity), p-ERK (#4370s, Cell signaling), ERK (#4695s, Cell signaling), NF-κB p65 (sc-8008, Santa Cruz), c-Jun (sc-44, Santa Cruz), and c-Fos (sc-52, Santa Cruz) antibodies on a shaker at 4 °C. The cytosolic and nuclear protein fractions were determined by WB analysis using anti-β-actin (A5441, Sigma Aldrich) and anti-C23 (sc-8031, Santa Cruz) antibodies as loading controls, respectively. Subsequently, the NC paper was rinsed with tris-buffered saline containing 0.1% Tween-20 (TBST) for 10 min three times at room temperature, incubated in horseradish peroxidase (HRP)-conjugated secondary antibodies, goat anti-mouse IgG or goat anti-rabbit IgG, for 1 h. After washing, the NC paper was then exposed to enhanced chemiluminescence (ECL) detection solution.

### 2.7. Immunoprecipitation (IP) Assay

The equal amounts (500 μg) of total protein samples prepared just as described above were incubated with 30 μL Protein A/G PLUS-Agarose (Santa Cruz Biotechnology, Santa Cruz, CA, USA) and 1 μg primary antibody, including E2F1, cdk-4 (sc-23896, Santa Cruz) or cdk-2 (sc-6248, Santa Cruz), then supplied on a rotating device overnight at 4 °C. Afterwards, the immunoprecipitates were centrifuged at 2500 rpm for 5 min at 4 °C to collect. The pellets were added 1 mL PBS to rinse three times with on a rotating device for 5 min each time. After washing finally, the pellets were re-suspended in SDS sample buffer, and subsequently boiled in 95 °C for 5 min. Furthermore, the immunoprecipitated proteins to detect antibodies against Rb, cyclin D1 (sc-426, Santa Cruz) or cyclin E (sc-481, Santa Cruz) were assayed by WB.

### 2.8. Wound Healing Assay

The A7r5 cells were seeded in 6-well plates (10^5^ per well). After the cells treated with or without GTIN had nearly grown to 100% confluency, the cell layer was wounded with a yellow pipette tip. Images of the cells were photographed each 24 h over a 3-day period after scraping in accordance with a previously studied procedure [25].

### 2.9. Transwell Migration and Invasion Assay

In the migration assay, 5 × 10^4^ A7r5 cells after treatment in serum-free medium were seeded into the upper chamber of a modified Boyden chamber (8-μm pore size; Neuro Probe, Cabin John, MD) [23], and a medium supplemented with 20% FBS, as a chemoattractant, was provided in the lower chamber. In the invasion assay, a layer of Matrigel (25 mg/50 mL; BD Bioscience) membrane was additionally coated on the upper chamber seeded with 10^5^ cells, and the medium with 20% FBS was added in the lower chamber. After incubating for 48 h, the Transwell chamber was picked out, and the medium in the chamber was discarded and rinsed with PBS. Afterwards, the cells invaded through the membrane were fixed with methanol for 30 min and stained with 10% Giemsa for 10 min. The cell image was captured and then counted under microscope.

### 2.10. Gelatin Zymography

Using gelatin zymography, the collected medium was subjected to 8% gel of SDS-PAGE containing 0.1% gelatin to assay the MMP-2/9 activities. Afterwards, the gels were rinsed with 2.5% Triton X-100 and incubated in a reaction buffer for 16 h at 37 °C. The gel was then stained with Coomassie brilliant blue R-250, as described previously [23].

### 2.11. Quantitative Reverse Transcription-Polymerase Chain Reaction (qRT-PCR)

To assess MMP-2/9 mRNA expression, total RNA of cell samples was isolated with TRIzol regent (Invitrogen, Thermo Fisher Scientific Inc., Waltham, MA, USA) in accordance with the manufacturer’s instructions. Firstly, the mRNA levels were detected by qRT-PCR applying a Bio-Rad iCycler system (Bio-Rad, Hercules, CA, USA), and normalized to an endogenous control, glyceraldehyde 3-phosphate dehydrogenase (GAPDH). The primers designed for real-time PCR were: GAPDH, forward 5′-TGGTGAAGGTCGGTGTGAAC-3′, and reverse 5′-GCTCCTGGAAGATGGTGATGG-3′; MMP-2, forward 5′-CTGACCCCCAGTCCTATCTGCC-3′, and reverse 5′-TGTTGGGAACGCCTGACTTCAG-3′; and MMP-9, forward 5′-CTTTGACAGCGACAAGAAGTGG-3′, and reverse 5′-GGCACTGAGGAATGATCTAAGC-3′.

### 2.12. Electrophoretic Mobility Shift Assay (EMSA)

Cellular nuclear extracts were prepared by utilizing NE-PER Nuclear and Cytoplasmic Extraction reagents (Thermo Fisher Scientific, Inc.). The DNA-binding abilities of NF-κB and AP-1 in nuclear extracts were detected by EMSA applying the Lightshift kit from Pierce (Rockford, IL, USA) [26]. The 10 μg nuclear protein was incubated with a buffer containing 10 mM Tris, 5 mM MgCl2, 50 mM KCl, 1 mM DTT, 2 μg poly (dI·dC), and 2 pmol biotin-labeled, double-stranded NF-κB or AP-1 oligonucleotides probes (Promega, Madison, WI, USA) at room temperature for 20 min. Subsequently, the protein-DNA complexes were separated on a 6% non-denaturing acrylamide gel, transferred to positively charged nylon membranes, and then UV cross-linked. Band shifts were visualized with a streptavidin-HRP, followed by ECL detection.

### 2.13. ROS Content Assay

Cellular ROS level was measured by utilizing 2′,7′-dichlorofluorescein diacetate (DCF-DA) fluorescent probes. The treated cells were collected, rinsed twice with 1 mL PBS, and subsequently incubated with 2 μM DCF-DA at room temperature and dark for 20 min. The fluorescence signal of ROS generation was measured using Muse™ Cell Analyzer. The values of each group were presented the relative to the fluorescence intensity of the starvation group.

### 2.14. Hydrogen Peroxide (H_2_O_2_) Assay

The cellular levels of H_2_O_2_ were determined by a H_2_O_2_ assay kit (Abcam, Cambridge, UK) in accordance with the manufacturer’s instructions. After the cell lysates were prepared, the lysate samples were added to the working solution containing OxiRed probe and HRP. In the presence of HRP, the OxiRed probe detects with H_2_O_2_ to generate a product that can be measured at OD570 nm by enzyme-linked immunosorbent assay (ELISA).

### 2.15. Statistical Examination

All quantitative data were shown as means ± standard deviation (SD) of three repeats from at least three independent experiments. Three or more separate experiments were carried out. For the concentration-response experiments of GTIN, one-way ANOVA with Dunnett’s post-hoc analysis was utilized to calculate the *p*-value for each concentration treatment compared to the untreated control. Regression was also applied to determine the *p*-value of the dependency of one parameter to dosage. A difference between experimental groups was statistically considered significant when the *p*-value was <0.05.

## 3. Results

### 3.1. Effect of GTIN on Abnormal VSMCs Proliferation

**Figure 1:**
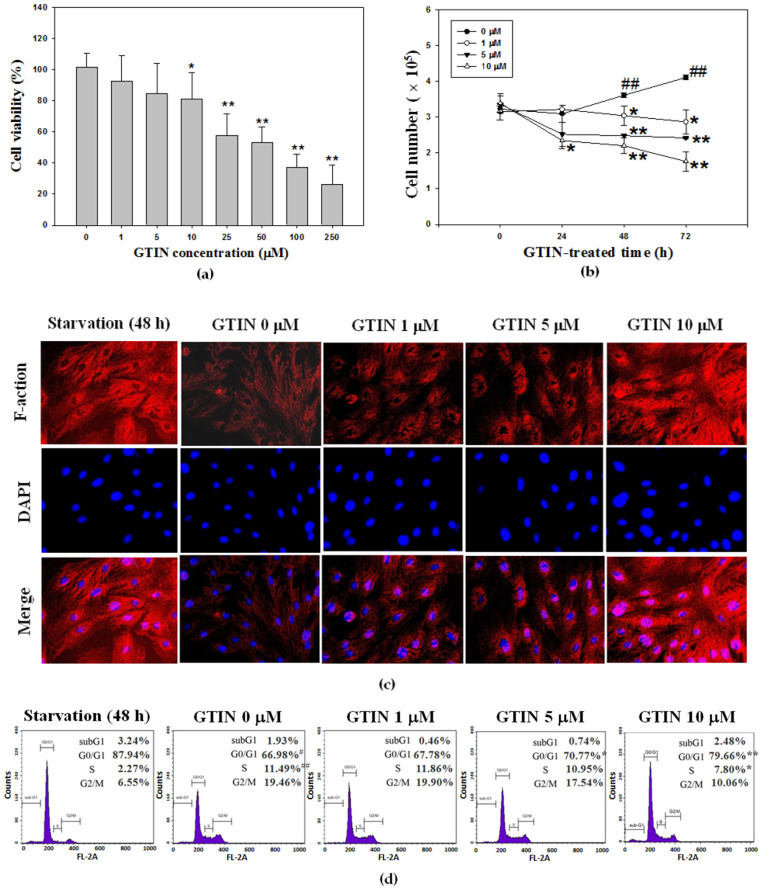
GTIN affected cell viability, proliferation, phenotype switch and cell-cycle progression in VSMCs (**a**) A7r5 cells were treated with GTIN at various doses (0–250 μM) for 24 h. Cell viability was determined by MTT method. The result was showed as mean ± SD of three repeats from at least three independent experiments. * *p* < 0.05, ** *p* < 0.01 compared with the untreated control via one-way ANOVA with Dunnett’s post-hoc analysis. (**b**) A7r5 cells were treated with GTIN at the indicated doses (1, 5 and 10 μM) for 24, 48 and 72 h. The cell growth curve was analyzed by applying trypan blue exclusion assay. The quantitative result was showed as mean ± SD of three repeats from at least three independent experiments. ^#^ *p* < 0.05, ^##^ *p* < 0.01 compared with the 0-h untreated control via one-way ANOVA with Dunnett’s post-hoc analysis. * *p* < 0.05, ** *p* < 0.01 compared with compared with the respective time point of the untreated control via one-way ANOVA with Dunnett’s post-hoc analysis. (**c**) A7r5 cells were pre-treated in DMEM supplemented with 0.5% FBS (starvation group) for 48 h, and then incubated with 10% FBS in the absence or presence of indicated doses of GTIN (1, 5 and 10 μM) for additional 48 h. The fixed cells were stained with TRITC-phalloidin (red) to detect F-actin, and counterstained with DAPI (blue) to represent cell nuclei. Representative images of VSMCs morphological alternation were obtained by fluorescence microscopy. (**d**) Under the same treatment condition, the cell-cycle distribution was assayed by using flow cytometery. The quantitative assessment of the percentage of each cell phase, including subG1, G0/G1, S and G2/M phase, in the cell-cycle distribution was revealed by PI dye. ^#^ *p* < 0.05, ^##^ *p* < 0.01, compared with the 48-h starvation group via student *t*-test. * *p* < 0.05, ** *p* < 0.01 compared with the untreated control via one-way ANOVA with Dunnett’s post-hoc analysis.

A preliminary screening by MTT assay was carried out to evaluate the effect of GTIN at various concentrations (from 1.0 to 250 μM) on A7r5 viability for 24 h, and it was indicated that a range between 1 and 10 μM of GTIN was nontoxic for A7r5 cells, while GTIN at dose higher than 25 μM inhibited the cell viability (Figure 1a). Hence 10 μM was utilized as the maximum dosage in the subsequent experiments. Furthermore, the growth curve of A7r5 cells was detected following the incubations with the indicated doses of GTIN every 24 h over a 3-day period. As shown in Figure 1b, the cell growth was diminished by 1, 5, and 10 μM GTIN in a dose- and time-dependent fashion, when compared to the untreated control. The result of cell growth curve declared that when GTIN at dosages above 1 μM for longer times (>24 h) were applied in the model of FBS-induced VSMCs proliferation, its anti-proliferative ability was more noticeable.

It is known that VSMCs dedifferentiation is connected with enhanced proliferative and migratory capacities [27]. Previous studies have reported that VSMCs in culture condition quickly fail to the contractile characteristics (contractile phenotype) and actively obtain the proliferating capacity (synthetic phenotype) showing features of less differentiated fibroblast-like cells upon progressive vascular lesions [28]. To evaluate the phenotype of A7r5 cells in the current culture condition and the impact of GTIN on the phenotypic switch of VSMCs, the expression of a contractile marker (also known as a differentiated marker), F-actin [29], was observed by ICC analysis. As presented in Figure 1c, a morphology change of A7r5 cells was short and round with low expression of F-actin upon FBS stimulation, while elongation and spindle with high expression of F-actin in starvation group (0.5% FBS for 48 h). In ICC analysis, the result was also found that GTIN treatments dramatically enhanced the expression of F-actin comparing the untreated control (Figure 1c), representing that GTIN corrected phenotypic switch and promoted the expression of differentiation marker in the FBS-model VSMCs. To further study the possible involvement of GTIN’s anti-proliferation in the modulation of VSMCs cell-cycle, the effects of GTIN (1, 5, and 10 μM) on A7r5 cells were detected by flow cytometry. In concordance with this VSMCs phenotypic transition, comparing with starvation group, a significant induction in the S phase was observed in the FBS-exposed cells, as the positive control in the following experiments. A 48 h-treatment of 5 and 10 μM GTIN resulted in an apparent accumulation of the cells in the G0/G1 phase from 70.77% to 79.66%, an increase of about 12% or more (Figure 1d).

### 3.2. Effect of GTIN on Cell-Cycle Regulatory Proteins in VSMCs

**Figure 2:**
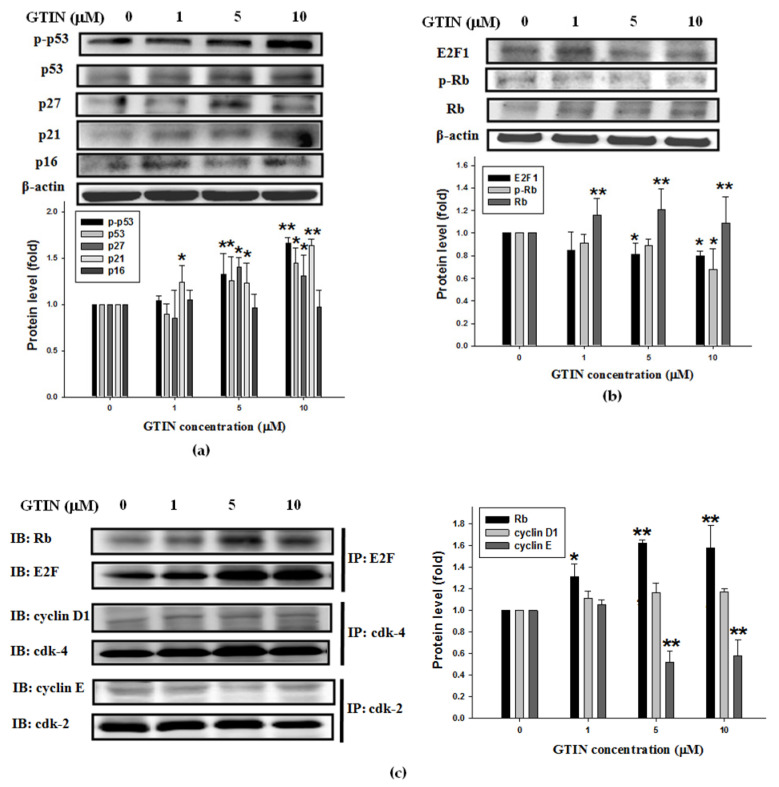
GTIN modulated cell-cycle regulatory factors in VSMCs. A7r5 cells were incubated with GTIN at the indicated doses (0, 1, 5 and 10 μM) for 48 h. The protein levels of p-p53, p53 and CDKIs, including p27, p21 and p16 (**a**), E2F1, Rb, p-Rb (**b**) and β-actin, as an internal control, were assayed by WB. (**c**) The expressions of Rb/E2F1, cyclin D1/cdk-4 and cyclin E/cdk-2 complexes were determined. The protein lysate was immunoprecipitated (IP) with anti-E2F1, cdk-4 or cdk-2 antibodies. The precipitated complexes were then analyzed for immunoblotting (IB) applying antibodies against Rb, cyclin D1 or cyclin E. All quantitative results were showed as mean ± SD of three repeats from at least three independent experiments. * *p* < 0.05, ** *p* < 0.01 compared with the untreated control via one-way ANOVA with Dunnett’s post-hoc analysis.

To investigate the underlying mechanisms of GTIN-arrested cell-cycle at G0/G1 phase, at 48 h the indicated doses of GTIN-treated A7r5 cells were subjected to WB analysis. The protein levels of CDKIs, including p27, p21, and p16, and their upstream factor p53 were first analyzed. Among them, p-p53, p53, p27, and p21 levels, not p16, were significantly increased by GTIN treatments at 5 and 10 μM (Figure 2a). In Go/G1 phase, a succession of cyclin/cdk complexes causes Rb hyperphosphorylation, releasing E2F1 from its sequestration together with Rb, and allowing E2F1 to transactivate genes essential for the S phase [30]. To determine the roles of Rb and E2F1 on the GTIN-blocked G0/G1 phase, the phosphorylated level of Rb and the expression of its complex with E2F1 were elucidated. The data indicated the protein level of Rb was markedly elevated, even though the phosphorylation of Rb was slightly reduced by 1, 5, and 10 μM GTIN treatments. After incubating with GTIN for 48 h, the GTIN treatment also inhibited significantly the protein of E2F1 (Figure 2b). Using IP assay, GTIN treatments induced the formation of Rb/E2F1 complex in A7r5 cells, confirming that an induction in Rb/E2F1 complex was correlated to a decline in the p-Rb level in the cell-cycle. To verify if GTIN induced the up-regulation of cdk inhibitors, contributing to inhibit cyclin/cdks activities, A7r5 cells were treated with GTIN at the indicated doses. As shown in Figure 2c, there was a significant reduction in expression of cyclin E/cdk-2 complex in the GTIN treatments, while no change in cyclin D1/cdk-4 complex. These results concluded that cyclin E/cdk-2 and Rb/E2F1 complexes participated in the GTIN-induced VSMCs cell-cycle arrest at G0/G1 stage.

### 3.3. Effect of GTIN on Abnormal VSMCs Migration

**Figure 3:**
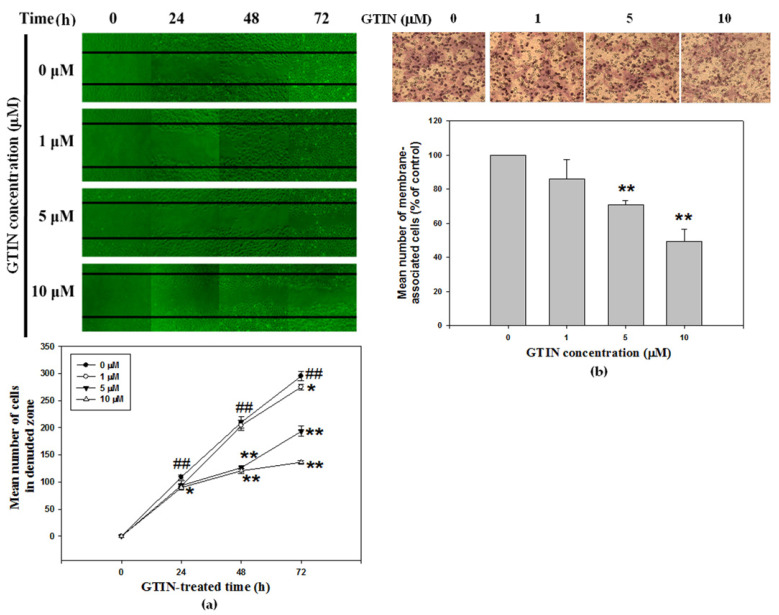
GTIN inhibited wound-healing and cell migration in VSMCs. (**a**) Monolayers of growth-arrested A7r5 cells treated with GTIN at the indicated doses (0, 1, 5 and 10 μM) were wound, and the cell numbers in the denuded zone were photographed and quantified for 0, 24, 48 and 72 h. The result was showed as mean ± SD of three repeats from at least three independent experiments. ^##^ *p* < 0.01 compared with the 0-h untreated control via one-way ANOVA with Dunnett’s post-hoc analysis. * *p* < 0.05, ** *p* < 0.01 compared with compared with the respective time point of the untreated control via one-way ANOVA with Dunnett’s post-hoc analysis. (**b**) A7r5 cells were treated with GTIN at the indicated doses (0, 1, 5 and 10 μM) for 48 h. The treated cells were subjected to assay for Transwell migration method. Images of the membrane-associated cells (purple parts) were then analyzed by Giemsa stain. The result was showed as mean ± SD of three repeats from at least three independent experiments. * *p* < 0.05, ** *p* < 0.01 compared with the untreated control via one-way ANOVA with Dunnett’s post-hoc analysis.

To determine the anti-migratory effect of GTIN in VSMCs, wound healing and Transwell assays were utilized. Wound healing assay detected the changes in cell migration by observing the cell numbers in the denuded zone under light microscopy. In the untreated control group, a gradual and apparent rise of the cell numbers in the denuded zone was noticed. On the contrary, the healing speed of the scratches in the GTIN-treated cells was lower than in the untreated control cells (upper panel, Figure 3a). As shown in Figure 3a (bottom panel), the quantitative data indicated that GTIN could dose- and time-dependently inhibit the migratory ability of A7r5 cells. To verify the results of the migration, the Transwell assay was further confirmed. The cell numbers of A7r5 in the groups of GTIN treatments that moved through the Transwell polycarbonate membrane were significantly and dose-dependently lower than that of cells in the untreated control group (Figure 3b). Similar to the result of migration, the invasiveness of the A7r5 cells in various groups was performed using the Transwell assay (data not shown).

**Figure 4:**
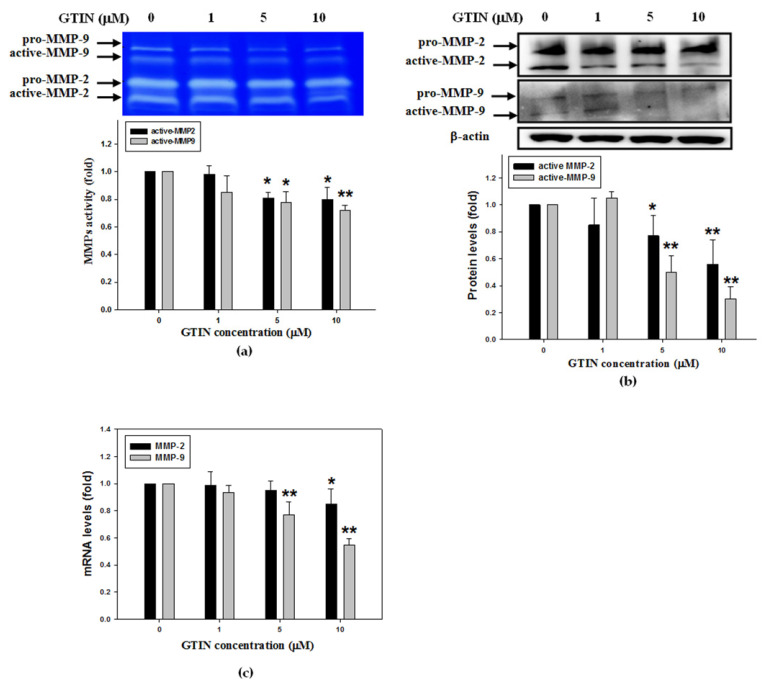
GTIN attenuated the MMP-2/9 activities and expressions in VSMCs. A7r5 cells were incubated with GTIN at the indicated doses (0, 1, 5, and 10 μM) for 48 h. (**a**) The medium of the treated cells was applied to gelatin zymography to determine the activities of MMP-2/9. The protein levels (**b**) and mRNA levels (**c**) of MMP-2/9 were respectively assayed by WB analysis and qRT-PCR. The results were showed as means ± SD of three repeats from three independent studies. * *p* < 0.05, ** *p* < 0.01 compared with the untreated control via one-way ANOVA with Dunnett’s post-hoc analysis.

In the above experiments, the inhibitory effect of GTIN on the migratory behavior of VSMCs was demonstrated, but its regulatory mechanism remains unknown. In order to explore the mechanism by which GTIN causes a decrease in cell migration, the activities of MMP-2/9, these factors are critical for the VSMCs migration, were further assayed using gelatin zymography. Figure 4a showed that MMP-2/9 activation was inhibited by GTIN in a concentration-dependent matter. Next, WB analysis indicated the protein levels of active-MMP-2/9 were increased significantly after GTIN treatments, specifically that of MMP-9 (Figure 4b). The GTIN mediated a reduction in the both protein levels, which was well in agreement with their mRNA levels as evidenced by applying qRT-PCR (Figure 4c), representing that GTIN down-regulated the transcriptional levels of MMP-2/9 to affect the expressions.

**Figure 5:**
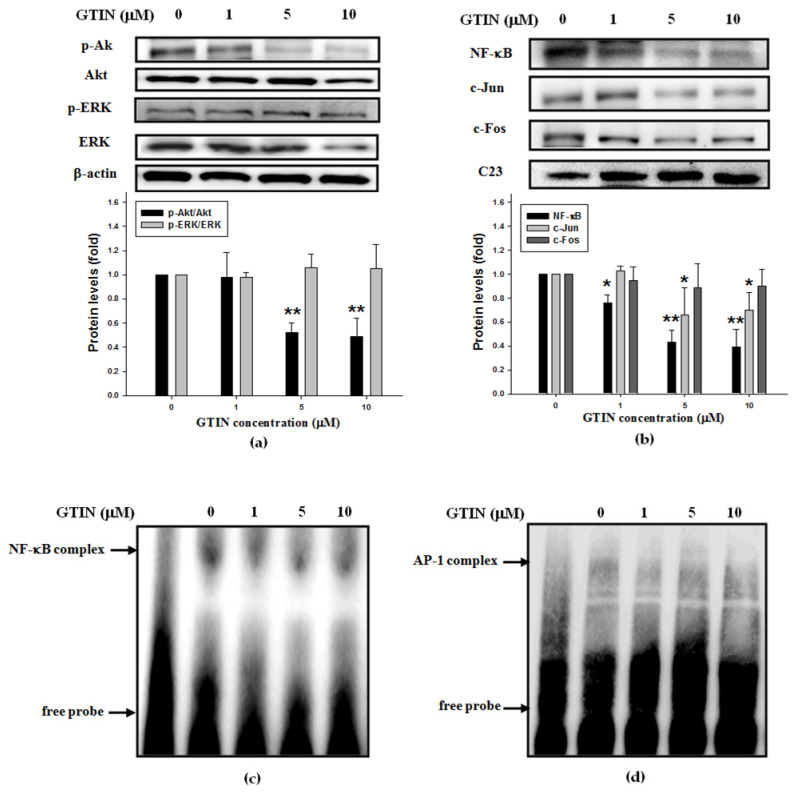
GTIN regulated Akt/NF-κB signaling pathways in VSMCs. A7r5 cells were incubated with GTIN at the indicated doses (0, 1, 5 and 10 μM) for 48 h. (**a**) The cytoplasmic levels of *p*-Akt, Akt, *p*-ERK, ERK and β-actin, as a cytosol internal control, were assayed by WB. (**b**) The nuclear levels of NF-κB, c-Jun, c-Fos and C23, as a nuclear internal control, were assayed by WB. The quantitative data was presented as means ± SD of three repeats from three independent studies. * *p* < 0.05, ** *p* < 0.01 compared with the untreated control via one-way ANOVA with Dunnett’s post-hoc analysis. (**c**) The DNA-binding abilities of nuclear NF-κB (**c**) and AP-1 (**d**) were analyzed using EMSA method. For the binding specificity controlling, Lane 1 represented the nuclear extracts reactive to free probe (the so-called unlabeled oligonucleotide). The results of EMSA were showed at least three independent experiments.

The migration of VSMCs has been shown to be regulated not only by the activities of MMP-9 [31], but also the up-regulation of Akt or ERK signaling pathway [32]. As shown in Figure 5a, GTIN reduced notably the phosphorylated level of Akt, but not that of ERK. Previous studies have also reported that promoter region of MMP-9 gene is located at several binding elements of transcription factors, such as NF-κB and AP-1 (a heterodimer of c-Jun and c-Fos), which serve as the downstream factors of Akt [19,23]. When compared with untreated control, GTIN treatments induced the significant nuclear expressions of the above transcription factors NF-κB p65, c-Jun, and c-Fos in A7r5 cells (Figure 5b). Furthermore, EMSA experiment also revealed that in A7r5 cells incubated with GTIN for 48 h, there were a marked reduction in the DNA-binding ability of the nuclear NF-κB (Figure 5c), and a minor decrease in that of AP-1 (Figure 5d). Therefore, it is possible the inhibitory effect of GTIN on the VSMCs migration was majorly conducted via down-regulating Akt/NF-κB that subsequently caused decreases in the expressions and activity of MMP-9.

### 3.4. Effect of GTIN on Cellular ROS Generation in VSMCs

**Figure 6:**
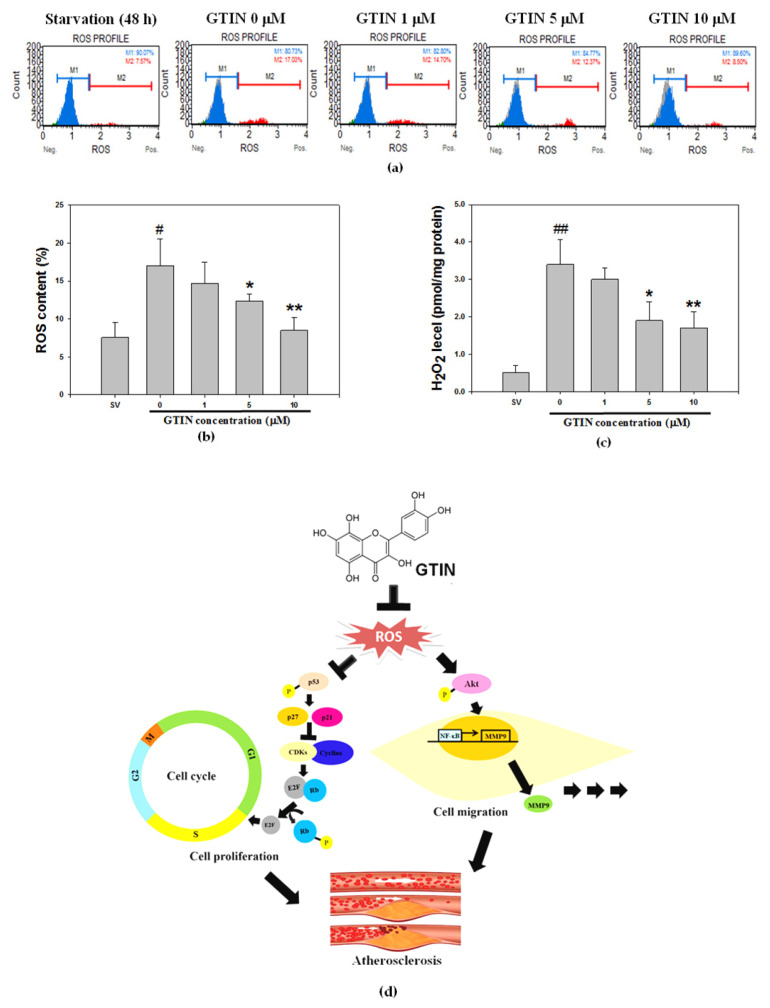
GTIN reduced cellular ROS generation in VSMCs. A7r5 cells were incubated with GTIN at the indicated doses (1, 5 and 10 μM) for 48 h. (**a**) The cells after treatment were labeled with DCF-DA, and ROS production was detected by measuring Muse™ Cell Analyzer. M1 and M2 respectively display DCF-negative and DCF-positive cell populations. (**b**) Quantitative assessment of the percentage of M2 was showed as means ± SD of three repeats from three independent studies. (**c**) The cellular level of H_2_O_2_ was measured using ELISA assay. The results were determined as means ± SD of three repeats from three independent studies. ^#^ *p* < 0.05, ^##^ *p* < 0.01, compared with the 48-h starvation (SV) group via student *t*-test. * *p* < 0.05, ** *p* < 0.01 compared with the untreated control via one-way ANOVA with Dunnett’s post-hoc analysis. (**d**) Schematic representation of the protective effects of GTIN against VSMCs dysfunctions. The in vitro effect of GTIN on cellular ROS generation, correcting the degree of abnormal VSMCs proliferation and migration was modulated by down-regulating Akt/NF-κB signaling-mediated MMP-9 production and up-regulating p53 signaling-mediated Rb phosphorylation that subsequently inhibited the development of atherosclerosis.

One review study has indicated that oxidative stress promotes the activity and expression of MMPs [11], and further studies also displayed the MMPs-mediated ECM remodeling is regulated by ROS [33]. To study the cellular oxidative stress diminished by GTIN in A7r5 cells, the contents of ROS production in different groups were assayed (Figure 6a). Comparing with the starvation group, the examination of cellular ROS levels revealed the FBS stimulation led to an increase in oxidative stress. Such an increase was reduced by the GTIN treatments in a dose-dependent fashion (Figure 6b), suggesting its antioxidant action. Under the same condition, GTIN also had an inhibitory effect on the H_2_O_2_ amount, the main kind of ROS, was coincided with the above result of ROS generation (Figure 6c). Taken together, these results suggested in vitro inhibitory effect of GTIN against ROS generation may correct the degree of abnormal VSMCs proliferation and migration by down-regulating Akt/NF-κB signaling-mediated MMP-9 production and up-regulating p53 signaling-mediated Rb phosphorylation that subsequently inhibited the development of atherosclerosis (Figure 6d).

## 4. Discussion

Hydroxylated flavones are one kind of flavonoids presented in fruits, vegetables, and plant-originated products (such as tea and red wine). Past findings have reported that hydroxylated flavones possess antimicrobial, antioxidant, anti-inflammatory, anti-cancer, and anti-atherosclerotic activities [34,35]. In the U.S.A. and U.K., the average daily human intake of the compounds has been estimated to be 1 g or more, which alleviates the risk of cardiovascular diseases like atherosclerosis [36]. The health-promoting effects of hydroxylated flavones intake have been attributed to their well-known radical-scavenging abilities—higher than ascorbic acid—and to the inhibitory properties on a wide range of protein enzymes [4]. Some well-known hydroxylated flavones have exhibited anti-atherosclerotic property, including resveratrol and quercetin [35,37]. Despite a lot of research associated with these flavonoids on the biological activity and structure–activity relationship in various cells, including tumor cells and immune cells [2,35,38], the anti-atherosclerotic study of hydroxylated flavones on the protection against VSMCs dysfunction, especially anti-proliferation and anti-migration in VSMCs, has not been fully evaluated. This in vitro study investigated the protective property of a natural hydroxylated flavone, GTIN, against the abnormal VSMCs proliferation and migration. To our understanding, this is the first study disclosing the protective effect of GTIN against the abnormal VSMCs dysfunction by inducing p53-arrested cell-cycle G0/G1 phase and inhibiting Akt/NF-κB-mediated MMP-9 expression.

Atherosclerosis is a chronic inflammatory process and has multistep participated in various soluble mediators, endothelial cells, macrophages, and VSMCs. Several soluble mediators, such as growth factors, cytokines, and chemokines, influence further the vascular wall by stimulating VSMCs proliferation and migration into foam cells [39]. FBS rich in growth factors able to stimulate the VSMCs proliferation and migration is historically applied for cultured cells [21,40]. A cellular model of the 48-h FBS incubation was utilized in this study to elucidate the effect of GTIN in VSMCs dysfunction. A7r5 cells were pre-cultured in medium with 0.5% FBS for 48 h to trigger the cells at resting stage (G0 phase) [21], as shown in this study’s results, and then incubated with 10% FBS together with or without GTIN at the indicated concentrations for additional 48 h. In our study, the FBS caused VSMCs proliferation, phenotype switch, and cell-cycle progression, entering S phase, a major event in atherosclerotic pathogenesis. The inhibitory effect of GTIN treatments was studied by MTT method, trypan blue exclusion assay, ICC, and cell-cycle examination, assays of which provided consistent results. This flavone, at dosages in a range of 1–10 μM, exhibited the protective effects, as evidenced by the results of decreases in the formazan formation from MTT uptake and growth rate, and increases in G0/G1 phase and even subG1 phases (apoptosis) in VSMCs. These findings revealed herein with VSMCs were positively correlated with the reports of Chen et al. (2013) [3], which indicated the GTIN showed potential in reducing intracellular lipid accumulation and foam cell formation in oxidized LDL-induced macrophage J774A.1 cells under non-cytotoxic doses (5, 10, and 20 μM). However, the protective property of GTIN against oxidized LDL-induced damage and apoptosis in human umbilical vein endothelial cells was attended at lower doses 0.1–0.5 μM [4]. A further study indicated the cardioprotective action of flavonoids on endothelial cells against apoptosis [41]. Jeong et al. (2005) has also reported the flavonoids have capability as potent antioxidants to diminish oxidized LDL-induced injury, and thus facilitate cell growth [42]. The results of our study demonstrate that GTIN’s protective effects on these different cellular models were examined in each assay, offering bifunctional results of GTIN at low and high doses. According to these results, it is persuading that GTIN potentially could be applied in the treatment of every stage in atherosclerosis.

VSMCs migration from vascular medial to intima contributes to the atherosclerotic progression [9]. In response to multiple stimuli, Akt is activated and phosphorylated followed plays a critical role in controlling cell migration and proliferation [11]. Activated Akt and its downstream factors, AP-1 and NF-κB, are required for the MMP-2/9 production and expression [26]. As expected, the GTIN-induced changes in the activities of MMP-2/9 coincided well with their protein and mRNA levels, especially in that of MMP-9. Next, to hypothesize the down-regulation of MMP-9 by GTIN is related to their transcription factors mediated MMP-9 expression. To reveal this hypothesis, the effect of NF-κB and AP-1 on their binding to promoter of MMP-9 gene in A7r5 cells was further examined. As illustrated in our data, GTIN significantly enhanced the DNA-binding ability of nuclear NF-κB to MMP-9 promoter region, while little affected that of AP-1, which was accompanied through decreases not only in the nuclear translocated level of the factor but also in the phosphorylated level of their upstream factor Akt. Consistent with past studies, this research confirmed the GTIN inhibited MMP-9 expression through the down-regulation of Akt/NF-κB signaling pathway, and consequently alleviated the VSMCs migration.

In addition, the Akt-mediated transcription factor NF-κB modulates cell-cycle progression in various cell types including VSMCs [43]. It also has been shown that Akt phosphorylates mouse double minute 2 homolog (MDM2), a p53-specific E3 ubiquitin ligase, on serine-166 and 186, promoting MDM2 translocation to the nucleus, where it down-regulates p53 function [44]. In this study, the result indicated the induction of p53 phosphorylation and expression by GTIN might be involved in blocking VSMCs proliferation. Besides, cell-cycle regulation by GTIN mediated decreases in expressions of cyclin E/cdk-2 and Rb/E2F1 complexes was mediated via up-regulation of the p53/p27 or p21 axis. Further studies are designed to examine whether treatment with GTIN can influence the proliferation of VSMCs via Akt/NF-κB or/and Akt/p53 signaling. Akt or NF-κB p65-specific siRNA was synthesized by in vitro transcription, screened out one efficacious siRNA in A7r5 cells, and utilized to perform the subsequent experiment. Many studies have also provided evidence that oxidative stress modulated by ROS is high association with the cell-cycle progression and MMPs-mediated ECM remodeling [11,33]. In this study, FBS induced the generation of intracellular ROS, especially in H_2_O_2_ content, and this might affect the overall signaling pathways of VSMCs dysfunction. While GTIN reversed the ROS generation with significance compared with untreated control, and it might lead to the inhibition of MMP-9 expression and cell-cycle program modulated by GTIN. Subsequent studies are required to explain this clearly.

Furthermore, there is a limitation in the application of FBS to induce VSMCs dysfunction in vitro [45], and very little research is available about which component of FBS influences VSMCs. To answer the question, a previous study utilizing primary rat VSMCs has demonstrated that 10% FBS was as effective as 20 ng/mL of platelet-derived growth factor (PDGF) to induce VSMCs proliferation, and further revealed that FBS and PDGF induced VSMCs proliferation by regulating the similar signaling pathways [46]. In vitro atherosclerotic studies, another model of tumor necrosis factor-alpha (TNF-α)-treated VSMCs usually has been used to mimic VSMCs dysfunction [23,39,47]. However, concerning the impact of TNF-α on VSMCs, there are inconsistent investigations [47]. Certain studies indicate that TNF-α does not stimulate the proliferation of VSMCs, conversely other reports declare that TNF-α causes VSMCs proliferation and migration via NF-κB-triggered transcription activation [47]. Several findings have demonstrated that TNF-α could promote VSMCs apoptosis through activation of caspase-3, however others revealed no apoptosis induction in the TNF-α-treated VSMCs [48]. Further literatures concluded the impacts of TNF-α on VSMCs may differ depending on cell phenotype and context [47,48]. The comparison of the effect of FBS verse PDGF and TNF-α in the induction of VSMCs dysfunction in vitro have been summarized in the Table S1. According to these past investigations, typical FBS rich in growth factors, especially PDGF and EGF, are able to stimulate the VSMCs dysfunction, and was applied in our studies. As expected, GTIN could show the capability to inhibit cell-cycle progression to S phase and cell migration in the TNF-α-treated A7r5 cells (Figure S1), similar to the results of FBS incubation. Further testing of GTIN in the model of TNF-α-induced VSMCs dysfunction is required to confirm these observations and declare the underlying mechanism. In the future, GTIN may be of great value in the development of therapeutic agents for atherosclerosis and other cardiovascular disorders.

## 5. Conclusions

The down-regulation of Akt/NF-κB signaling-mediated MMP-9 production and the up-regulation of p53 signaling-mediated cell-cycle arrest were mediated by the in vitro action of GTIN against ROS generation, controlling the balance of abnormal VSMCs proliferation and migration. GTIN plays an inhibitory role in VSMCs dysfunction by multiple mechanisms: suppression of cell proliferation, cell-cycle progression, migration, matrix degradation, and oxidative stress.

## Data Availability

Data is contained within article and supplementary materials.

## Supplementary Materials

The following are available online at https://www.mdpi.com/article/10.3390/antiox10091357/s1, Table S1: Comparison of the effect of FBS verse PDGF and TNF-α in the induction of VSMCs dysfunction in vitro, Figure S1: GTIN inhibited cell-cycle progression and wound-healing in TNF-α-treated VSMCs.
