# Supplementary material for: Anti-Atherosclerotic Effect of Gossypetin on Abnormal Vascular Smooth Muscle Cell Proliferation and Migration
Source: Antioxidants (Basel). 2021 Aug 26;10(9):1357. doi: 10.3390/antiox10091357 (PMC8470489; doi:10.3390/antiox10091357)
Supplement: Supplementary file 1 [file antioxidants-10-01357-s001.zip › antioxidants-1309018-supplementary.pdf]

Supplementary Data

Table S1- Comparison of the effect of FBS *verse* PDGF and TNF-α in the induction of VSMCs dysfunction *in vitro*

Inducer / Induction of VSMCs dysfunction	**Fetal bovine serum (FBS)**	**Plateled-derived growth factor (PDGF)**	**Tumor necrosis factor-alpha (TNF-α)**
Effective dosage	10%	20-100 ng/mL	10-100 ng/mL
Proliferative response [a]	+++ [b]	++ [b] - +++ [c]	+ [b]
Migratory response [d]	↑ [e]	↑	↑
Apoptosis activation [f]	NA [g]	NA	↑
Signaling pathway [h]	P53, p21, p27, Rb/E2F1, Akt, NF-κB, STAT3, AP-1	Raf-1/Rb, PI3K/Akt, Ras/Erk, p38, NF-κB, STAT3	Raf-1/Rb/E2F1, PI3K/Akt, ERK, p38, NF-κB, AP-1
Suitable model	Cholesterol-laden atherosclerotic plaques, and neointimal hyperplasia (restenosis)	VSMCs differentiation, vascular remodeling, and aortic aneurysm model construction	Inflammatory vascular diseases, such as atherosclerotic intimal hyperplasia and preeclamptic hypertension
Reference	Peppel et al. 2005<br>Wu et al. 2009<br>Davis et al. 2012<br>Chen et al. 2013<br>Lin et al. 2021	Peppel et al. 2005<br>Wu et al. 2009<br>Davis et al. 2012	Chau et al. 2004<br>Peppel et al. 2005<br>Davis et al. 2012<br>Chou et al. 2019

[a] Effect of different inducers on proliferative response in primary human aortic VSMCs (AoSMCs) was analyzed by bromodeoxyuridine (BrdU) incorporation assay. [b] Relative induction of proliferation is scored on an arbitrary 1–3 (+- +++) scale, based on fold/basal measurements derived from data of Davis et al. 2012. [c] According to another previous studies reported by Wu et al. (2009) and Chen et al. (2013) using rat VSMCs, the result showed that 10% FBS was as effective as 20 ng/mL PDGF to promote cell proliferation. [d] Effect of 10% FBS *vs.* 20 ng/mL PDGF on primary rat VSMCs migratory response by wound healing and transwell assays, while effect of PDGF *vs.* TNF-α in the same dosage (100 ng/mL) on primary rabbit VSMCs by transwell assay. [e] ↑ represents "elevated". [f] Chau et al. (2004) has shown that TNF-α could induce apoptosis in VSMCs via caspase-3 activation. [g] NA represents "not applicable". [h] Davis et al. (2012) has revealed that PDGF and TNF-α have very different signaling intermediates, and their downstream functions require activation of protein kinases (PI3K/Akt, Erk and p38) and transcription factors (NF-κB, STAT3 and AP-1) in the induction of VSMCs dysfunction.

Figure S1

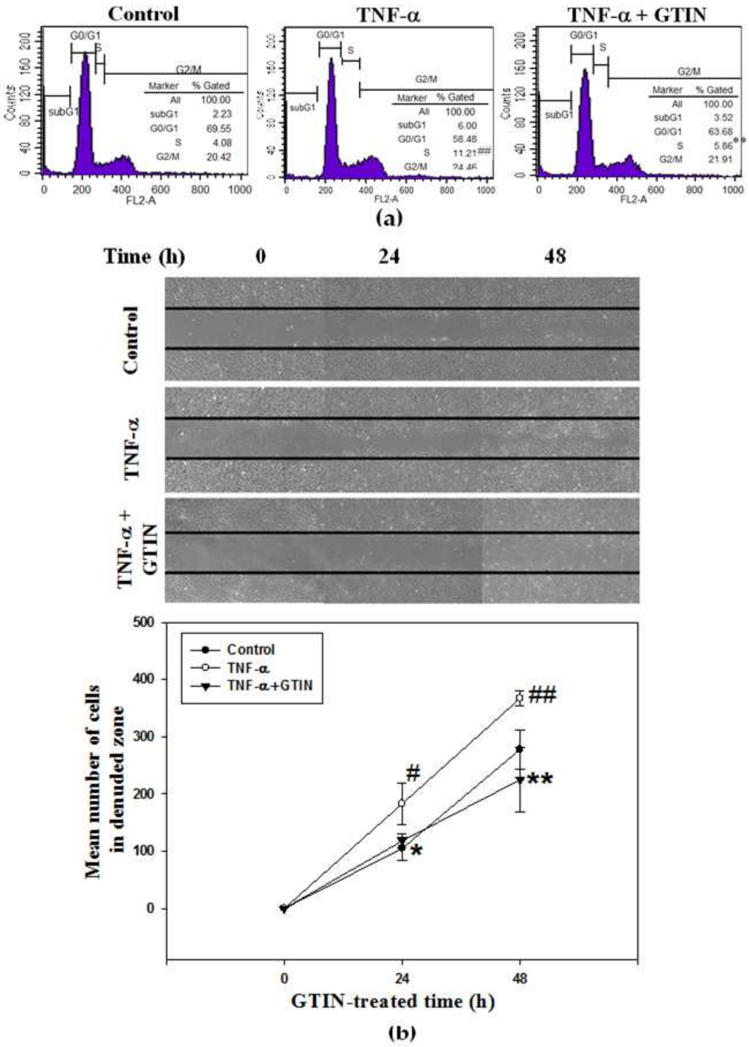

Figure S1. GTIN inhibited cell-cycle progression and wound-healing in TNF-α-treated VSMCs. (a) A7r5 cells were treated with or without TNF-α (10 ng/mL) in the absence or presence of 10 μM GTIN for 24 h. the cell-cycle distribution was assayed by using flow cytometery. The quantitative assessment of the percentage of each cell phase, including subG1, G0/G1, S and G2/M phase, in the cell-cycle distribution was revealed by PI dye. ##p< 0.01, compared with the control via student t-test. **p< 0.01 compared with the TNF-α-treated group via student t-test. **(b)** Monolayers of growth-arrested A7r5 cells treated with or without TNF-α (10 ng/mL) in the absence or presence of 10 μM GTIN were wound, and the cell numbers in the denuded zone were photographed and quantified for 0, 24 and 48 h. The data was showed as mean ± SD of three repeats from at least three independent experiments. #p< 0.05, ##p< 0.01 compared with the 0-h control via student t-test. *p< 0.05, **p< 0.01 compared with compared with the respective time point of TNF-α-treated group via student t-test.
